# Reconstruction of Nasal Skin Cancer Defects with Local Flaps

**DOI:** 10.1155/2011/181093

**Published:** 2011-06-07

**Authors:** A. C. Salgarelli, P. Bellini, A. Multinu, C. Magnoni, M. Francomano, F. Fantini, U. Consolo, S. Seidenari

**Affiliations:** ^1^Department of Head and Neck Surgery, Unit of Maxillofacial Surgery, Modena and Reggio Emilia University, Via del Pozzo 71, 41100 Modena, Italy; ^2^Department of Dermatology, Head and Neck Skin Cancer Service, Modena and Reggio Emilia University, Via del Pozzo 71, 41100 Modena, Italy

## Abstract

Reconstruction of nasal defects must preserve the integrity of complex facial functions and expressions, as well as facial symmetry and a pleasing aesthetic outcome. The reconstructive modality of choice will depend largely on the location, size, and depth of the surgical defect. Individualized therapy is the best course, and numerous flaps have been designed to provide coverage of a variety of nasal-specific defects. We describe our experience in the aesthetic reconstruction of nasal skin defects following oncological surgery. The use of different local flaps for nasal skin cancer defects is reported in 286 patients. Complications in this series were one partial flap dehiscence that healed by secondary intention, two forehead flaps, and one bilobed flap with minimal rim necrosis that resulted in an irregular scar requiring revision. Aesthetic results were deemed satisfactory by all patients and the operating surgeons. The color and texture matches were aesthetically good, and the nasal contour was distinct in all patients. All scars were inconspicuous and symmetrical. No patient had tenting or a flat nose.

## 1. Introduction

The most common site of facial skin cancer is the nose (25.5%), because of its cumulative exposure to sunlight [1–3]. When dealing with primary non-melanoma nasal skin cancers, the most important goal is to obtain a tumor-free patient. Several studies have outlined the surgical parameters necessary for the excision of primary nonmelanoma skin cancers [4–6]. Well-defined primary basal cell carcinomas (BCCs) less than 2 cm in diameter should be excised with 4.0-mm margins to obtain a 95% cure rate [5]. Primary squamous cell carcinomas (SCCs) require 4.0-mm margins for low-risk tumors and 6.0 mm margins for high-risk tumors (≥2.0 cm; >II histological grade; nose, lip, scalp, ears, eyelids; invasion into the subcutaneous tissue) to obtain a 95% cure rate [4, 6]. For these tumors, Mohs micrographic surgery offers improved cure rates, as it is a technique that allows for complete microscopic control of tumor removal in addition to superior tissue preservation. The Mohs technique described in 1941 is based on the concept of excising skin cancer layer by layer and examining horizontally cut specimen sections to view the entire surgical margin. The disadvantages of the Mohs technique are that it is labor intensive, time consuming, and quite dependent on the skills of not only the Mohs surgeon/pathologist but also the histotechnician who prepares the specimens. In addition, high cost has been a criticism of Mohs surgery in the literature [7]. After tumor-free margins on frozen section have been established, reconstruction of the surgical wound can be performed with confidence. 

Given the vital functions of the nose in everyday life, it is extremely important that the reconstruction of facial defects preserves the integrity of complex facial functions and expressions, as well as facial symmetry and a pleasing aesthetic outcome. When planning the reconstruction of surgical defects, a surgeon must carefully consider a number of characteristics unique to the nose, including the inherent structural complexity of the nose, with convex and concave surfaces in close proximity, the symmetry of the nose, the limited laxity of the nasal skin, and the sebaceous composition of distal nasal skin. Finally, the function of the nose must be maintained by preserving or replacing the bony and cartilaginous framework and the mucosal lining and by never compromising a patent airway. Re-establishing the framework in nasal reconstruction is critical to achieving both form and function. As the options for producing these results may be limited in some cases, familiarity with a variety of flaps is essential [8, 9]. 

Here, we review our experience with nasal reconstructions. Surgical defects in each subunit were usually repaired in a predictable and reproducible fashion. The results of a review of 286 patients with surgical defects of the nose following excision of skin malignancies are presented.

## 2. Patients and Methods

Consecutive patients (*n* = 286) referred for excision of nonmelanoma skin cancers on the nose, from 2002 to 2009, were included. They comprised 167 males and 119 females, aged 42 to 92 years, who were followed for between 6 months and 7 years. The most common skin cancers of the nose in these patients were basal cell carcinomas (190 cases) and squamous cell carcinomas (96 cases). After a shave biopsy confirmed a malignancy, all patients underwent excision of the skin cancer with margins appropriate for the type, behavior, and size of the lesion. All specimens underwent histological examination. Frozen histological sections were examined for lesions of more than 1 cm in diameter. All patients underwent primary reconstruction after excision of the malignancy. Immediately after tumor excision, all wounds were managed by primary closure; local skin flaps, including bilobed double transposition flap, nasolabial flap, modified nasalis flap, or forehead flap; or a combination of reconstructive modalities to preserve the nasal topographic aesthetic subunits [10–15]. The excisions were performed under local anesthesia or local anesthesia plus intravenous sedation, except in those patients whose wounds were closed with a forehead flap, who received general anesthesia and constant monitoring of flap vascularity by the surgeon. 

The cosmetic outcome was evaluated at 6 months after surgery. The location, depth, and size of the skin defect; the quality of the adjacent skin; the reconstruction choice; and the cosmetic result were recorded. 

The reconstructive modality of choice depends largely on the location, size, and depth of the surgical defect.

## 3. Direct Closure

Direct elliptical closure undermining the supraperichondrial or supraperiosteal plane was usually used for defects up to 1 cm in diameter. Upper nonsebaceous nasal areas were most amenable to direct closure. 

Wide undermining is crucial for sufficient skin laxity and must be extended to the nasal facial junction. When significant advancement has been achieved, the margins of the surgical defect may be readily approximated under minimal tension. The resulting surgical defect is then closed with 5-0 Vicryl buried vertical mattress sutures, keeping the sutures within the subcutaneous tissue and deep reticular dermis [16]. Special care should be taken to keep the sutures deep, because placement that is too superficial may leave permanent dimples. The buried sutures should approximate the edge closely enough that the top layer of the running cuticular sutures is under no tension. Owing to its advantages of simplicity, fewer suture lines, and fewer complications, primary closure has long been used to avoid the limitations inherent in reconstruction using flaps or grafts. However, the skin over the lower third of the nose has limited mobility and cannot readily be recruited for closure of anything but small defects. Therefore, if primary closure will lead to unacceptable results, more complex wound reconstruction should be considered.

## 4. Bilobed Flap

**Figure 3:**
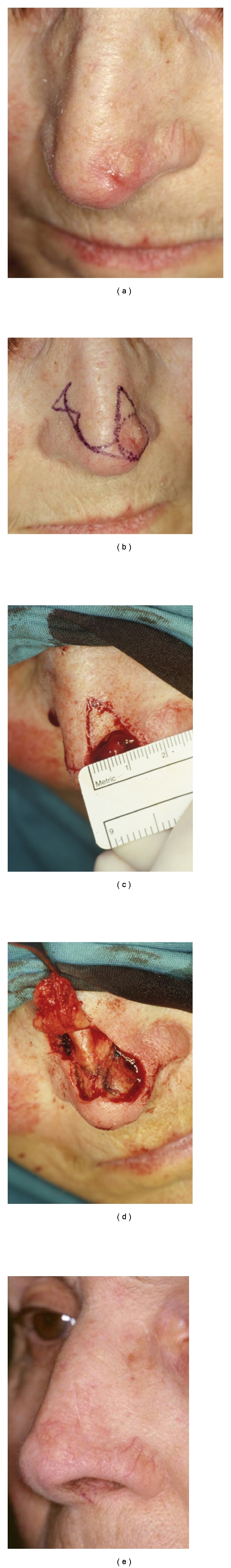
(a) BCC involving the lateral tip of the nose. (b) Zitelli's flap design. (c) Tumour resection. (d) Intraoperative view: double transposition flap. (e) Nasal appearance two years after surgery.

The Zitelli's bilobed flap is one of the most useful flaps for nasal reconstruction [10, 11]. It is a simple double transposition flap (Figure 3) and is designed to move more skin, without deformation, over a larger distance than would be possible with a single transposition flap in the same location. This is the repair of choice for defects located between 0.5 and 1.5 cm of the distal and lateral aspect of the nose, particularly defects involving the lateral tip, supratip, or tissue near the tip [10, 14, 16]. On the lower third of the nose, where the skin is least mobile, the bilobed flap allows the surgical site to be filled with nearby skin and matched for color and texture; it then allows for repair of the secondary defect with another well-matched flap from a nearby donor site. The initial lobe should be the same size as the defect, but the secondary lobe may be slightly smaller to allow for donor site closure with minimal distortion. The angle of transposition is approximately 45–50° for each lobe. The defect, flap, and donor site should be widely undermined in the periosteal and perichondrial planes to facilitate transposition without distortion of the nasal tissue and to reduce pin cushioning. An adequate Burow's triangle must be removed from the pivot point to eliminate bunching and dog-ear formation. It can be designed with its base medial or lateral. Flaps based laterally on the side wall of the nose are most useful for reconstruction of defects near the nasal tip, whereas medially based flaps are more useful for repair of alar defects. Bilobed flaps are the best for small defects in the tip or ala [17, 18]. In cases with defects located between 1.5 and 2.0 cm of the distal and lateral aspect of the nose, particularly those involving the nasal tip or alar lobules, more complex wound reconstructions should be considered.

## 5. Modified Nasalis Flap

**Figure 1:**
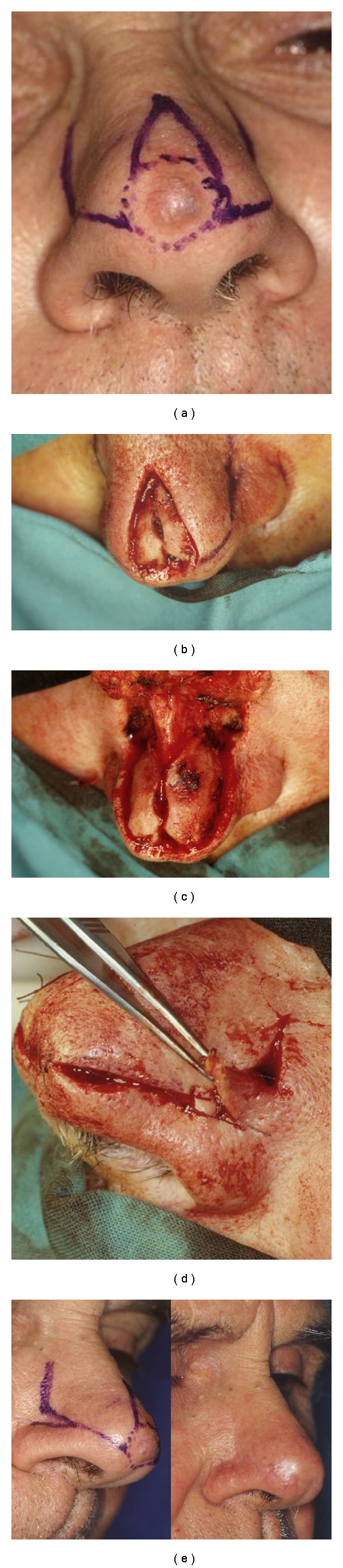
(a) BCC involving nasal tip: flap design. (b) Tumour resection. (c) Intraoperative view: modified bilateral nasalis flap. (d) Interpositional flap from the alar groove. (e) Nasal appearance before and one year after surgery.

Aesthetic reconstruction of the nasal tip and supratip areas following skin tumor excision is a challenge. The tip is the aesthetic focal point of the nose, and irregularities in color, texture, and thickness are easily noted [9]. The modified nasalis flap provides an additional option for reconstruction of this difficult area (Figure 1) and has been extremely useful for the closure of central and lateral nasal tip and supratip defects of up to 2.0 cm in diameter [14]. It is a simple transposition flap based on the angular artery that rotates toward the midline and nasal tip and leaves donor scars located in the nasojugal and alar creases. 

Owing to our dissatisfaction with the original technique as presented in the literature, we prefer using a modified nasalis flap as a bilateral flap for coverage of central tip defects, even when they are not large [14]. In this way, we minimize nasal distortion and create symmetrical scars, providing better aesthetic results [19]. Following tumor excision, an incision is made in the superior alar sulcus, extending to the nasojugal fold. A backcut is then made in the nasojugal fold, parallel to the nasolabial fold. The transposition flap, including the nasalis muscle, and an interpositional flap from the lateral alae are elevated completely at the perichondrial and deep subcutaneous levels, respectively. The arterial branches of the nasalis muscle should be identified and preserved during elevation of the transposition flap. As the nasalis flap is transposed in an anterior and caudal direction, a midline dog-ear that requires resection is created. The interpositional flap from the alar groove rotates in an opposite and cephalad direction to fill the donor defect in the nasojugal fold in order to minimize scarring for closure of wider defects and to maintain the definition of the nasojugal fold [19]. Donor scars are well concealed, and the nasal contour is minimally altered. The wide flap base minimizes postoperative edema and has prevented the pincushion deformity common to small local transposition and advancement flaps. The flap can be raised in a single stage under local anesthesia, and late revisions have not been required.

## 6. Nasolabial Flap

**Figure 2:**
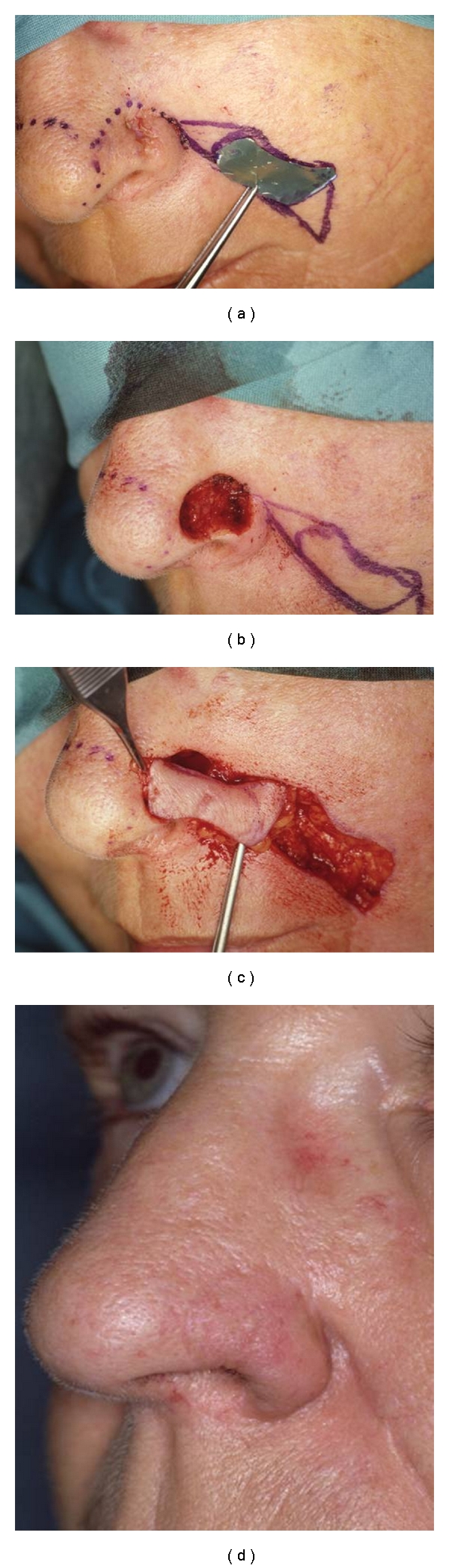
(a) Nasolabial flap design. (b) Tumour resection. (c) The flap is transferred to the recipient site. (d) Nasal appearance two years after surgery.

In the case of defects with diameters between 1.5 and 2.0 cm and involving the alar lobules, a nasolabial transposition flap is useful for reconstruction in this difficult area (Figure 2) [12, 13, 17]. The nasolabial flap is a superiorly based transposition flap that makes use of the abundant cheek skin. A small amount of excess tissue that matches the nose in color and texture lies near the melolabial fold, but its underlying fat has a strong tendency to contract. The melolabial fold can supply enough skin to resurface the ala, and the contractility of the nasolabial flap can be used to simulate the round, expected bulge of the normal ala [17, 20]. Abundant tissue is usually available in the melolabial area, and the maximum width of the flap is limited only by the amount of cheek tissue that can be used in the flap and still effect primary closure of the donor site in the melolabial sulcus. 

Furthermore, the skin is usually free of hair and has an excellent blood supply from the branches of the facial artery. 

As the first step in this procedure, the exact pattern of the contralateral normal ala is determined just superior to the melolabial sulcus. The flap is designed as an interpolation flap in which the final scar of donor site closure lies exactly in the melolabial sulcus. The flap is traced 1 mm larger in all dimensions to allow for postoperative contraction. The inset is thinned distally, leaving only 1-2 mm of subcutaneous tissue in the area of the inset. The donor site is closed by undermining adjacent cheek skin and advancing it, inferiorly and medially. Closure of the donor defect before closure of the primary defect brings the base of the flap closer to the nose, thereby facilitating subsequent closure of the primary defect with minimal wound closure tension. 

Three weeks later, the flap inset is partially elevated, and excess subcutaneous and scar tissue are sculpted from the alar base, lip, and cheek join; in the same procedure, cartilage grafts to prevent scar contraction can be performed as necessary. The normal concavity of the nasofacial sulcus can be re-established, using an absorbable suspension suture placed between the undersurface of the dermis of the flap and the periosteum of the nasal bone or maxilla. After further 3 weeks, the pedicle is divided. The residual pedicle, which served as a vascular carrier, is discarded, and the cheek is closed by advancement, so that the final scar lies exactly in the alar facial sulcus and melolabial sulcus.

## 7. Forehead Flap

**Figure 4:**
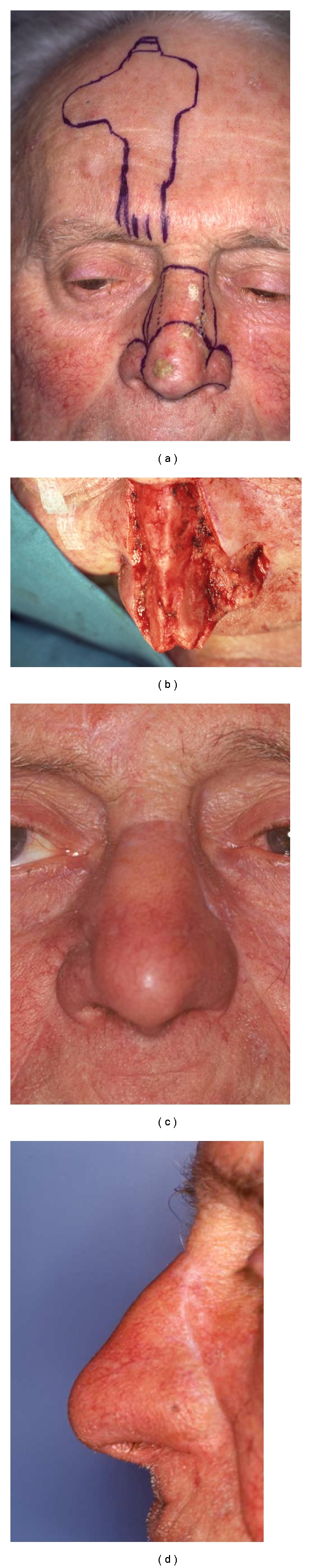
(a) Multifocal nasal skin cancer: flap design. (b)Skin defect after tumour resection. (c) Nasal appearance one year after surgery: frontal view. (d) Nasal appearance one year after surgery: lateral view.

In general, defects greater than 2.5–3 cm in diameter are difficult to close with a nasolabial flap. Local transposition flaps are precluded, and distant tissue such as a forehead flap will usually be required (Figure 4) [13, 15, 21]. In our experience, the pedicle flap most commonly used on the nose is the median forehead flap. It is a two-stage, advanced procedure for reconstruction of large and deep surgical defects of the distal nose, especially where the cartilage framework has been sacrificed. Its base lies close to the defect, between the medial brow and medial canthus. First, an exact three-dimensional pattern is made of the defect. Typically, this is designed on the contralateral normal side or on an ideal model. Forehead skin does not contract, and so the pattern is designed exactly. The forehead flap is excised to the periosteum at the base of the flap, to the upper parts of the subcutaneous tissue, in order to avoid the axially and vertically oriented feeding arteries. 

The success of this flap depends on the preservation of its vascular pedicle, the supratrochlear artery, and the thinning of the subcutaneous tissue from the distal flap before suturing it into the wound. Forehead skin is used only for nasal coverage and not for adjacent lip or cheek defects. The central vertical component is employed to resurface the dorsum, tip, and columella, and its lateral wings are used to wrap around the ala and curve into the nostril floor and alar sill, in cases requiring total nasal resurfacing. Redundant tissue is removed from the forehead in both the horizontal and vertical directions when closing the donor site. This facilitates primary closure of the vertical component as an inconspicuous vertically oriented paramedian scar and the lateral wings as scars that lie in the natural transverse wrinkle lines of the forehead. 

Three weeks later, the flap inset is partially elevated, and excess subcutaneous and scar tissue are sculpted; in the same procedure, cartilage grafts to prevent scar contraction can be performed as necessary. The pedicles remain intact for approximately 3 weeks, allowing the ingrowth of blood vessels from the recipient site. Then, it is divided, and the unused part is returned to the forehead. 

Techniques for the reconstruction of larger defects involving multiple subunits and the adjoining cheeks remain a matter of debate. In these cases, a combination of reconstructive modalities is necessary to preserve the nasal topographic aesthetic subunits [15].

## 8. Results

A total of 286 patients who underwent nasal reconstruction after ablative skin cancer surgery (190 basal cell carcinomas, 96 squamous cell carcinomas) were treated with the procedures described above. The nasal reconstruction distribution for the 286 patients included 94 bilobed flaps, 17 modified nasalis flaps, 15 nasolabial transposition flaps, 71 forehead flaps, 6 combinations of reconstructive modalities, and 83 cases of direct elliptical closure (Table 1). The bilobed flap was the most commonly used flap on the nose; of the 203 wounds repaired with a local flap, 94 were repaired with a bilobed double transposition flap, and most of these were located in the lower third of the nose. 

Defects in all nasal topographic units were treated, with some patients having defects involving multiple subunits. In such cases, a combination of reconstructive modalities are necessary to preserve the nasal topographic aesthetic subunits. The mean age of the patients at the time of surgery was 67.3 years. There were 167 men and 119 women. 

The follow-up period ranged from 6 months to 7 years (mean, 38.5 months). 

Comorbidities included diabetes, hypertension, smoking, and previous histories of nasal skin cancer ablation. In the 83 patients who underwent direct elliptical closure there were 12 minor complications such as superficial infection or hematoma with secondary healing. In the 203 patients who underwent nasal reconstruction with a local flap, there was no flap failure. Complications in this series were one case of partial flap dehiscence that healed by secondary intention, two forehead flaps, and one bilobed flap with minimal rim necrosis that resulted in an irregular scar requiring revision. 

Aesthetic results were deemed satisfactory by all patients and the operating surgeons.The color and texture matches were aesthetically good and the nasal contour was distinct in all patients. All scars were inconspicuous and symmetrical. No patient had tenting or a flat nose. We had a total of 9 (0.32%) recurrences on 286 patients: two on 83 direct elliptical closure, three on 94 bilobed flaps, one on 15 nasolabial flap and three on 71 isolated forehead flaps.

## 9. Discussion

Aesthetic and functional reconstruction of full-thickness soft-tissue nasal defects involves many options. Although the topographic nasal subunit principle of Burget and Menick [15] is important in preoperative analysis and planning of the reconstruction, other aesthetic considerations such as skin texture, color, and contour are also crucial [9, 21]. A balance must be achieved among these various factors and the patient's medical condition, adjacent tissue availability, skin history, and expectations [9, 22]. 

A patient's medical history can significantly affect the reconstruction plan, by forcing all treatment into a monitored operating room environment. Diabetics and smokers should be warned about potential skin necrosis, and a different plan of reconstruction or the delay of flaps may be necessary in these patients. Skin history is important, and patients with scars from previous nasal cancers may require a modified treatment plan. In these patients, a flap may be used to incorporate a past scar; on the other hand, scar tissue may impede the blood supply to a flap. Finally, patient expectations can influence reparative concerns. For example, a young woman may want optimal cosmetic results, whereas an older man may not have as many cosmetic concerns [21]. 

The reconstructive modality of choice will depend largely on the location, size, and depth of the surgical defect. Nevertheless, reconstructive plans should be customized and not be based solely on the size or location of the defect. Individualized therapy is the best course, and numerous flaps have been designed to provide coverage of a variety of nasal-specific defects. We recommend that reconstructive techniques be selected according to the anatomical nasal subunits to be restored, whenever possible [23]. Direct elliptical closure with undermining in a supraperichondrial or supraperiosteal plane was typically used for defects up to 1 cm in diameter. Upper nonsebaceous nasal areas were most amenable to direct closure. 

A skin graft is generally not considered the ideal replacement for nasal skin, in particular for the thick, sebaceous skin of the nasal tip, ala, lower sidewalls, or dorsum. The basic concern with using a skin graft is the resultant patchwork appearance caused by color mismatch and contour defects. Nevertheless, superficial defects larger than 1 cm will be treated with full-thickness skin graft successfully [24]. 

When peripheral concerns such as prior skin history and smoking are minimal and the only desire is an excellent cosmetic result, flaps are a superior way to close defects. 

Esser designed the first bilobed flap in 1918 and applied it to the reconstruction of defects of the nasal tip [25]. In 1989, Zitelli adapted the design of Esser's bilobed flap by reducing its rotation angles, and then it is one of the most useful flaps for nasal reconstruction [10, 11]. 

It is designed to move more skin over a larger distance than would be possible with a single transposition flap in the same location. Thus, it is the repair of choice for defects located within 0.5 and 1.5 cm of the distal and lateral aspects of the nose, particularly those involving the lateral tip, supratip, or ala near the tip [10, 15, 18]. In the lower third of the nose, where the skin is least mobile, a bilobed flap allows the surgical site to be filled with nearby skin that is matched for color and texture, and then allows for repair of the secondary defect with another well-matched flap from a nearby donor site. The nose tip is the aesthetic focal point of the nose, and irregularities in color, texture, and thickness are easily noted [8, 9, 12, 17]. 

The modified nasalis flap provides an additional option for reconstruction of this difficult area [14, 19]. This flap has proven to be extremely useful for the closure of central and lateral nasal tip and supratip defects of up to 2.0 cm in diameter [21]. 

In cases of defects with a 1.5- to 2.0-cm diameter that involve the alar lobules, a nasolabial transposition flap is useful for reconstruction of this difficult area [12, 13]. The nasolabial flap is a superiorly based transposition flap that makes use of abundant cheek skin. 

A small amount of excess tissue that matches the nose in color and texture lies near the melolabial fold, and its underlying fat has a strong tendency to contract. The melolabial fold can supply enough skin to resurface the ala, and the contractility of the nasolabial flap can be used to simulate the round, expected bulge of the normal ala [20]. The donor site scar from the melolabial transposition flap is relatively easy to camouflage in the natural expression lines of the face. 

Larger defects often require a forehead flap. Forehead skin with a width of 4 or more cm can be harvested without tissue expansion [7, 15, 26]. The pedicle flap most commonly used on the nose is the median forehead flap. It is a two-stage advanced procedure for the reconstruction of large and deep surgical defects of the distal nose, especially where the cartilage framework has been sacrificed. The depth of the defect governs the choice of material for reconstruction. When bone or cartilage is exposed, a local or distant flap is appropriate, according to the size of the defect. Without a skeletal framework, the soft tissue of the cover and lining would collapse, impairing the airway and limiting projection. Re-establishing a framework in nasal reconstruction is of paramount importance for retaining form and function and for maintaining optimal three-dimensional reconstruction. However, when the underlying nasal support is missing and a cartilage framework must be restored with primary cartilage grafts, a local flap is no longer applicable. A delicate cartilage reconstruction would be distorted or collapse under the tension of wound closure. In such circumstances, a distant flap (nasolabial or forehead flap) would be required. In replacing the missing normal cartilaginous framework of each unit, the primary cartilage grafts should be as wide as the defect, and not only as wide as the missing cartilage framework, in order to provide a rigid skeleton for support, projection, and contour and to brace the reconstruction against the force of myofibroblast contraction. 

Even a perfectly designed and executed reconstructive surgery needs appropriate postoperative care and followup to optimize the final outcome. Informing patients that their cooperation with postoperative instructions will contribute to optimal cosmetic results tends to increase patient compliance.

## Figures and Tables

**Table 1:** Surgical wound management of the nose ( 286 cases).

Mode	Patients	%Treated
Direct elliptical closure	83	29,1%
Bilobed flap	94	32,9%
Modified nasalis flap	17	5,9%
Nasolabial transposition flap	15	5,2%
Forehead flap	71	24,8%
Forehead flap + nasolabial flap	6	2,1%
